# Loss of Gαq impairs regulatory B-cell function

**DOI:** 10.1186/s13075-018-1682-0

**Published:** 2018-08-24

**Authors:** Yan He, Xiaoqing Yuan, Yan Li, Chunlian Zhong, Yuan Liu, Hongyan Qian, Jingxiu Xuan, Lihua Duan, Guixiu Shi

**Affiliations:** 1 Department of Rheumatology and Clinical Immunology, The First Affiliated Hospital of Xiamen University, Xiamen, China; 2 Ningbo City Medical Treatment Center Lihuili Hospital, No. 57 Xingning Road, Ningbo, 315000 China

**Keywords:** Gαq, Regulatory B cells, IL-10, Regulation

## Abstract

**Background:**

Recent studies have shown a crucial role of Gαq in immune regulation, but how Gαq modulates regulatory B-cell (Breg) function is still unclear. We address this here.

**Methods:**

CD19^+^IL-10^+^ Bregs of wild-type (WT) and *Gnaq*^*−/−*^ mice were analyzed by flow cytometry after stimulation by lipopolysaccharide. The WT and *Gnaq*^*−/−*^ Bregs were isolated and cocultured with WT CD4^+^CD25^−^ T cells in the presence of T-activator, and the proliferation of T cells and differentiation of regulatory T cells (Tregs) were analyzed by flow cytometry. We used inhibitors of PI3 kinase (PI3K), extracellular regulated protein kinases 1/2 (Erk1/2), and p38 mitogen-activated protein kinase (p38 MAPK) to detect the pathways involved in the regulation of Gαq on Breg differentiation, which were confirmed by western blot analysis. Furthermore, the expression level of Gαq was assessed by quantitative real-time PCR in peripheral blood mononuclear cells (PBMCs) from healthy controls and rheumatoid arthritis patients. The frequency of CD19^+^CD24^hi^CD38^hi^ B cells in PBMCs was detected by flow cytometry, and the association of the Gαq mRNA expression level and the frequency of CD19^+^CD24^hi^CD38^hi^ B cells was analyzed by Spearman test.

**Results:**

The differentiation of CD19^+^IL-10^+^ Bregs was inhibited in the *Gnaq*^*−/−*^ mice. In addition, Gαq depletion showed an impaired suppressive function of Bregs on T-cell proliferation, which might be due to the decreased Treg expansion. Mechanically, our data demonstrated that the PI3K, Erk1/2, and p38 MAPK signaling pathways were required for regulation of Gαq on Bregs, and blockage of these signaling pathways impaired Breg differentiation. Consistent with our previous studies, we also found a decreased frequency of CD19^+^CD24^hi^CD38^hi^ Bregs in rheumatoid arthritis patients. As expected, a significantly positive correlation was investigated between CD19^+^CD24^hi^CD38^hi^ Bregs with Gαq mRNA expression.

**Conclusions:**

Our results indicate that Gαq plays a critical role in the differentiation and immunosuppression of Bregs, and it may provide a new therapeutic target for autoimmune diseases.

**Electronic supplementary material:**

The online version of this article (10.1186/s13075-018-1682-0) contains supplementary material, which is available to authorized users.

## Background

B cells are best known for their capacity to produce antibodies. In addition, they also exert a variety of other functions during the immune response, including antigen presentation and production of various cytokines, which are involved in the early and late stages of T-cell-mediated immune responses [1]. However, B-cell-deficient mice were observed to be susceptible to experimental autoimmune encephalitis (EAE), and to be unable to recover from it [2]. Furthermore, adoptive transfer of IL-10^+^ B cells can suppress inflammation of EAE [3]. A new population of B cells, regulatory B cells (Bregs), has increasingly gained attention for restraining inflammation [4, 5]. Bregs can suppress the differentiation of T helper 1 (Th1) and T helper 17 (Th17) cells, and promote regulatory T-cell (Treg) induction [6, 7]. It was also reported that Bregs support the maintenance of invariant nature killer T (iNKT) cells [8]. Bregs have been shown to inhibit autoreactive and pathogen-driven immune response mainly through the production of interleukin-10 (IL-10), interleukin-35 (IL-35), and transforming growth factor beta (TGF-β) [9]. Until now, the production of immune-suppressive cytokine IL-10 was thought to be a hallmark of Breg function [10]. In some human autoimmune diseases, it has been reported that Breg function is impaired and does not prevent the development of human autoimmune diseases, such as RA [7], relapsing–remitting multiple sclerosis [11], systemic lupus erythematosus (SLE) [12], and so on [13]. However, the mechanism of impaired Breg function in autoimmune diseases remains unclear.

The heterotrimeric guanine nucleotide-binding proteins (G proteins) are important signal transducers, which when attached to the cell surface plasma membrane receptors, G protein-coupled receptors (GPCRs), can communicate with signals from a large number of hormones, neurotransmitters, chemokines, sensory stimuli, and autocrine and paracrine factors. The heterotrimeric G proteins are composed of three subunits (α, β, and γ subunits) that cycle between inactive and active signaling states in response to guanine nucleotides [14, 15]. On the basis of downstream signaling targets of α subunits, these α subunits are divided into four classes: Gαi/0, Gαs, Gαq/11, and Gα12/13. Gαq is a member of the Gαq/11 subfamily encoded by GNAQ [16]. Gαq is ubiquitously expressed in mammalian cells and nearly 40% of all GPCRs rely upon Gαq family members to stimulate inositol lipid signaling [15]. It is well known that Gαq plays an essential role in the nervous system, endocrine system, and cardiovascular system [16–20]. Many studies have also established the physiological importance of Gαq in the immune system. A previous study showed that Gαq-deficient (*Gnaq*^*−/−*^) mice exhibited impaired eosinophil recruitment to the lung after antigenic challenge, probably due to an impaired production of granulocyte macrophage colony stimulating factor (GM-CSF) by resident airway leukocytes [21]. Our previous study reported that *Gnaq*^*−/−*^ dendritic cells were defective in migrating from the skin to draining lymph nodes after fluorescein isothiocyanate sensitization, and *Gnaq*^*−/−*^ monocytes were defective in migrating from the bone marrow into inflamed skin after contact sensitization [22]. The functional involvement of Gαq in TCR-induced immune responses was also investigated [23]. In addition, *Gnaq*^*−/−*^ chimeras could spontaneously develop manifestations of systemic autoimmune disease with high titer antinuclear antibody and inflammatory arthritis, which was observed in our previous study [24]. In humans, our previous work also showed that Gαq mRNA expression was decreased in peripheral blood lymphocyte cells (PBMCs) and T cells from SLE patients compared to that from healthy individuals. What is more, the Gαq expression in T cells from SLE patients was associated with disease severity, the presence of lupus nephritis, and expression of Th1, Th2, and Th17 cytokines [25]. We also found that B cells from mice lacking the Gαq subunit of trimeric G proteins have an intrinsic survival advantage over normal B cells, suggesting that Gαq is critically important for maintaining control of peripheral B-cell tolerance induction and repressing autoimmunity [24]. Whether Gαq regulates Breg function is still unknown.

In this study, we found a critical role of Gαq in Breg differentiation and *Gnaq*^*−/−*^ Bregs showed an impaired suppressive function on T-cell proliferation. Our human data also showed that the decreased frequency of Bregs showed a significantly positive correlation with Gαq mRNA expression in RA patients. Taken together, our work reveals a novel function of Gαq in regulating Breg function.

## Methods

### Patients and controls

Peripheral blood was obtained from 34 RA patients and 24 healthy controls from the inpatient clinic of the Department of Rheumatology, The First Affiliated Hospital of Xiamen University, Xiamen, China. The criteria used for RA diagnosis were based on those of the American Rheumatism Association (1987) [26] and the new criteria from the ACR/EULAR (2010) [27]. Gαq mRNA expressions were detected by RT–PCR, the frequency of CD19^+^CD24^hi^CD38^hi^ B cells in PBMCs was detected by flow cytometry, and the association of Gαq mRNA expression level and frequency of CD19^+^CD24^hi^CD38^hi^ B cells was studied. The clinical characteristics of the RA patients are summarized in Table 1. Informed consent was obtained from all recruits to this study. This study was approved by the Ethics Committee of the First Affiliated Hospital of Xiamen University in accordance with the World Medical Association Declaration of Helsinki.

**Table 1:** Demographic data and clinical characteristics of RA patients in the study

Characteristic	RA (*n* = 34)	HC (*n* = 24)
Age (years), mean (range)	56 (34–78)	45 (29–68)
Sex (*n*), female/male	26/8	17/7
CRP (mg/L), mean (range)	30.35 (0.5–75.1)	
ESR (mm/h), mean (range)	47.29 (7–117)	
RF (IU/ml), mean (range)	223.35 (9.69–991)	
Anti-CCP (RU/ml), mean (range)	52.50 (1–200)	
Tender joint count, mean (range) of 68 joints	6.35 (0–26)	
Swollen joint count, mean (range) of 68 joints	4.47 (0–22)	

*RA* rheumatoid arthritis, *HC* healthy controls, *CRP* C-reaction protein, *ESR* erythrocyte sedimentation rate, *RF* rheumatoid factor, *anti-CCP* anti-cyclic citrullinated peptide antibody

### Animals

All experimental procedures involving mice were approved by the institutional animal care committee of Xiamen University. C57BL/6 J (B6) mice were purchased from Xiamen University Laboratory Animal Center. C57BL/6 J (B6) mice and *Gnaq*^*−/−*^ (*n* > 8 backcrossed to C57BL/6 J) mice were bred in Xiamen University Laboratory Animal Center. The mice used in this study were 6–8 weeks age.

### Cell isolation

The purification of CD4^+^CD25^−^ T cells from the spleen of mice was performed using the CD4^+^CD25^−^ T Cell Isolation Kit (Miltenyi Biotec, Bergisch-Gladbach, Germany), LS Columns (Miltenyi Biotec), and MidiMACS™ Separators (Miltenyi Biotec) according to the manufacturer’s instructions. Briefly, 1*10^7^ cells from the mice spleen were stained with biotin-antibody cocktail in buffer (PBS/2 mM EDTA/0.5% BSA) for 5 min at 4 °C. After that, the anti-Biotin MicroBeads and CD44 MicroBeads were sequentially added and incubated for 10 min at 4 °C.Cells were washed, centrifuged, and resuspended in 0.5 ml of buffer, and applied onto the column. The CD4^+^CD25^−^ T cells in flow-through fluids were collected. The isolation of B cells and Tregs from the spleen of mice was performed using the corresponding Pan B Cell Isolation Kit II (Miltenyi Biotec) and the CD4^+^CD25^+^ Regulatory T cell Isolation Kit (Miltenyi Biotec) according to the manufacturer’s instructions. Purity of the target cells was > 90% in all experiments assessed by flow cytometry. CD1d^hi^CD5^+^ B cells were isolated using a MoFlo High-Performance Cell Sorter (Beckman Coulter, Fullerton, CA, USA) with purities of 90–95%. Human PBMCs were isolated from 4 ml sodium heparin-treated venous blood samples by Ficoll density-gradient centrifugation using Lymphoprep™ (Axis-Shied PoC AS, Oslo, Norway). Washed and resuspended, the PBMCs were cryopreserved for future real-time polymerase chain reaction.

### Cell culture

Purified B cells were planted in complete RPIM 1640 with 2.05 mM l-glutamine (GE Healthcare Life Sciences, Logan, UT, USA) supplement with 10% fetal bovine serum (PAN Seratech, Aidan Bach, Germany) and maintained in standard cell culture environment (95% humidity, 5% CO_2_ at 37 °C). For Breg induction, B cells were stimulated with LPS (*Escherichia coli* 0111:B4; Sigma-Aldrich, St. Louis, MO, USA) (10 μg/ml) for 48 h. PMA (50 ng/ml), ionomycin (250 ng/ml) (Sigma-Aldrich), and brefeldin A (10 μg/ml) (BD Biosciences, San Jose, CA, USA) were added for the last 5 h of culture before flow cytometry. For analysis of CD4^+^ T-cell proliferation and Treg differentiation, purified LPS-induced Bregs from the spleen of WT mice or *Gnaq*^*−/−*^ mice and sorted CD4^+^CD25^−^ T cells from the spleen of WT mice were 1:1 cocultured and activated with Dynabeads™ Mouse T-Activator CD3/CD28 (Life Technologies AS, Oslo, Norway) at a bead-to-cell ratio of 1:2 in 200 μl medium in a 96-well U-bottom plate for 72 h.

### Antibodies

Anti-human antibodies included: anti-CD19-FITC from BD Biosciences; anti-CD24-PE (ML5) and anti-CD38-PE /Cy7 (HIT2) from Biolegend (San Diego, CA, USA); and Human FcR Binding Inhibitor from eBioscience (San Diego, CA, USA). Anti-mouse antibodies included: anti-CD4-FITC (RM4–5), anti-CD19-PE/Cy5, anti-CD19-APC (6D5), anti-CD25-APC (3C7), anti-IL-10-PE (JES5-16E3), PE Rat IgG2b, κ Isotype Ctrl (RTK4530), and anti-PD-L1-APC (10.F.9G2) from Biolegend; anti-CD1d-PE (1B1), anti-CD5-FITC (53–7.3), anti-CD16/CD32 (mouse Fc block), anti-Annexin-V-APC, and 7-AAD from BD Biosciences; and anti-CD25-PE/Cy5.5 (PC61.5), anti-Foxp3-PE (NRRF-30), anti-TLR4-Alexa Fluor® 488(UT41), and anti-FasL-FITC (MFL3) from eBioscience. Phosho-p38 MAPK (Thr180/Tyr182) (28B10) mouse mAb, p38 MAPK (D13E1) XP® rabbit mAb, phosho-p44/42 MAPK (Erk1/2) (Thr202/Tyr204) (D13.14.4E) XP® rabbit mAb, p44/42 MAPK (Erk1/2) (137F5) rabbit mAb, phosho-PI3K p85 (Tyr458)/p55 (Tyr199) antibody, PI3K p85α (6G10) mouse mAb, phospho-STAT1 (Thr701) (58D6) rabbit mAb, STAT1 (D1K9Y) rabbit mAb, GAPDH (14C10) rabbit mAb, β-Tubulin (9F3) rabbit mAB, anti-mouse IgG, HRP-linked antibody, anti-rabbit IgG, and HRP-linked antibody were obtained from Cell Signaling Technology (MA, USA); and anti-MyD88 antibody was obtained from Abcam (Cambridge, UK).

### Real-time polymerase chain reaction

Total RNA was extracted from PBMCs with TriPure Isolation Reagent (Roche Diagnostics GmbH, Mannheim, Germany) and the concentration of RNA was determined by measuring the absorbance at 260 nm in a UV–Vis spectrophotometer (Quawell, San Jose, CA, USA). Reverse transcription was performed by the Bio-Rad Systems (Bio-Rad, Hercules, CA, USA) according to standard protocols using the Transcriptor First Strand cDNA Synthesis Kit (Roche Diagnostics GmbH). The expression level of Gαq was measured by real-time quantitative PCR. β-actin was simultaneously amplified and used as an internal control. The primer sequences were as follows: β-actin forward, 5′-AGAAAATCTGGCACCACACC-3′; β-actin reverse, 5′-AGAGGCGTACAGGGATAGCA-3′; Gαq forward, 5′-GTTGATGTGGAGAAGGTGTCTG-3′; and Gαq reverse, 5′-GTAGGCAGGTAGGCAGGGT-3′. Amplification was performed with the 7500 Real Time PCR Systems (Applied Biosystems, CA, USA). Gene expression levels were normalized by comparing to β-actin and relative expression was calculated by the2^–ΔΔCt^ method.

### Enzyme-linked immunosorbent assay

The concentration of mouse IL-10 (BD Biosciences), IL-35 (Wuhan Huamei, China), TGF-β, IL-23 (Invitrogen, Carlsbad, CA, USA), and IL-6 (R&D, Minneapolis, MN, USA) were measured using commercially available ELISA kits according to the manufacturer’s instructions. Absorbance at 450 nm was measured with an ELISA microplate reader (Molecular Devices, Sunnyvale, CA, USA).

### Flow cytometry

Fc receptors were blocked with mouse Fc block and the dead cells were detected using Fixable Viability Dye eFlour™ 506 or 510 (eBioscience) before cell surface staining. For Breg staining, CD19-FITC or PE/Cy5, CD24-PE, CD38-PE/Cy7, CD1d-PE, and CD5-FITC mAbs were used. For intracellular IL-10 staining, cells were stained with CD19-PE/Cy5 or APC mAbs. Cells were washed, fixed with IC Fixation Buffer (eBioscience), permeabilized with Permeabilization Buffer (eBioscience), and stained with IL-10-PE. For Treg staining, cells were stained with combinations of CD4-FITC and CD25-PE/Cy5.5 or APC mAbs, fixed and permeabilized with Fixation/Permeabilization solution (eBioscience) and Permeabilization Buffer, and stained for detection of intracellular Foxp3-PE mAbs. For apoptotic cell detection, cells were washed twice with cold PBS and then resuspended in 1× Binding Buffer (BD Biosciences), and then the cells were stained with CD4-FITC, APC Annexin-V, and 7-AAD and incubated for 15 min at RT in the dark. Last, 400 μl of 1× Binding Buffer was added. Data were acquired using Cytomic FC500 or Cytoflex (Beckman Coulter) and analyzed using CXP Analysis and Cytexpert (Beckman Coulter).

### Western blot analysis

Single-cell suspensions were lysed after stimulation in cOmplete Lysis-M (Roche Diagnostics GmbH) containing protease inhibitor cocktail (Roche Diagnostics GmbH) and phosphatase inhibitor cocktail (Roche Diagnostics GmbH) for 10 min with gentle shaking. The lysates were centrifuged at 14,000 × *g* for 15 min, and frozen at − 80 °C until use. For western blotting assays, the protein concentrations were determined using the BCA Protein Assay Kit (Thermo Scientific, Rockford, IL, USA), and equal amounts of protein (20 μg) per lane were separated by 10% SDS-PAGE gels and transferred to PVDF membranes. Membranes were blocked and then probed with primary antibodies against p-p38 MAPK (1:1000), p-Erk1/2 (1:2000), p-PI3K (1:1000), p-STAT1 (1:1000), or MyD88 (1:1000) at 4 °C overnight. After washing, the membranes were incubated with the appropriate HRP-conjugated secondary antibodies for 1 h at room temperature. After extensive washing, signals were visualized using the chemiluminescent HRP substrate system (Millipore, Billerica, MA, USA). Band quantification was performed on the Molecular Imager® ChemiDoc™ XRS+ system with Image Lab™ Software (Bio-Rad, Hercules, CA, USA). Thereafter, membranes were stripped with stripping buffer before reprobing with anti-p38 MAPK (1:1000), Erk1/2 (1:1000), PI3K (1:1000), and STAT1 (1:1000) to ensure equal loading. GAPDH or β-Tubulin was also detected as the loading control. The level of protein phosphorylation was normalized to the loading control (total protein).

### Statistical analysis

All data were obtained from at least three independent experiments and shown as mean ± standard deviation (SD). All data were analyzed using GraphPad Prism 5.01 software (GraphPad, San Diego, CA, USA). Statistical significance was determined by Student’s *t* test and the Mann–Whitney *U* test. Correlation was analyzed using Spearman’s test. A probability value of *p* < 0.05 was considered statistically significant.

## Results

### Gαq regulates Breg differentiation

**Fig. 1:**
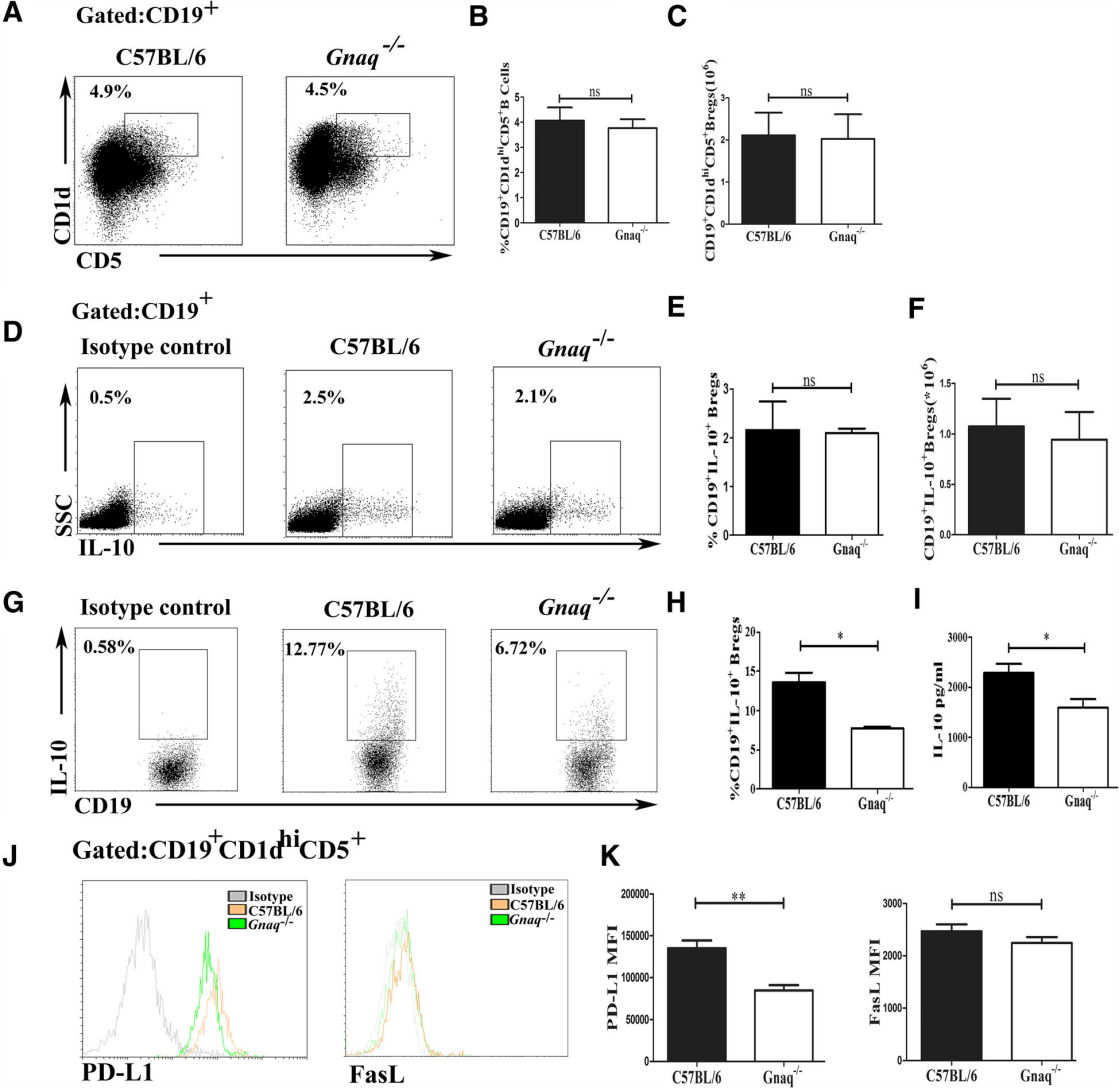
Loss of Gαq limited differentiation of CD19^+^IL-10^+^ Bregs. **a–f** Splenic cells isolated from *Gnaq*^*−/−*^ mice and WT littermates and subjected to flow cytometry analysis. Splenic cells stained with anti-mouse CD19, CD1d, and CD5 after PMA, ionomycin, and BFA stimulation, followed by intracellular staining with IL-10. CD19-positive cells gated for analysis of CD1d^hi^CD5^+^ cells (**a**, representative images; **b**, statistical analysis). IL-10-positive cells in CD19^+^ gate also analyzed (**d**, representative images; **e**, statistical analysis). Absolute number of CD19^+^CD1d^hi^CD5^+^ and CD19^+^IL-10^+^ cells also quantified (**c**, **f**). **g**, **h** B cells isolated from spleen of WT and *Gnaq*^*−/−*^ mice and stimulated with LPS for 48 h, and then PMA, ionomycin, and BFA added for last 5 h. After culture, cells stained with anti-mouse CD19, followed by intracellular staining with IL-10 and analysis by flow cytometry (**g**, representative images; **h**, statistical analysis). Results represent mean ± SD per group (*n* = 6–8 mice/group). Student’s *t* test analyzed statistical difference. Data representative of three independent experiments. **i**–**k** Purified B cells from spleens of WT and *Gnaq*^*−/−*^ mice stimulated with LPS for 48 h, then culture supernatants harvested and subjected to analysis of IL-10 production by ELISA (**i**), and cells collected to analyze PD-L1 and FasL expression. **j** Representative histograms show PD-L1 and FasL expression on Bregs from WT mice (red line), *Gnaq*^*−/−*^ mice (green line), and isotype control (gray line). Mean fluorescence intensity (MFI) of PD-L1 and FasL expression also recorded by flow cytometry (**k**). Data presented as mean ± SD (*n* = 6–8), and Student’s *t* test performed to analyze statistical difference. Data representative of three independent experiments. *p < 0.05, **p < 0.01. IL interleukin, ns not significant, SSC side scatter

We and others have reported that Gαq plays a critical role in immune disorders via regulating immune cell function. Recently, a crucial role of Bregs has been described in many studies. Nevertheless, whether Gαq regulates Breg function remains unknown. The *Gnaq*^*−/−*^ mice were used to address this question. The flow cytometry analysis showed that there was no difference between WT and *Gnaq*^*−/−*^ mice on the percentage of CD19^+^CD1d^hi^CD5^+^ Bregs (Fig. 1a, b) and CD19^+^IL-10^+^ Bregs (Fig. 1d, e), both of which are considered Breg markers in mice [9]. Additionally, no marked difference in the absolute number of Bregs was observed (Fig. 1c, f). Interestingly, a significantly higher expression of IFN-γ and IL-17 was observed in *Gnaq*^*−/−*^ CD19^+^CD1d^hi^CD5^+^ Bregs (see Additional file 1: Figure S1A–D). Although increased production of IFN-γ by *Gnaq*^*−/−*^ Bregs was observed, the STAT1 phosphorylation after LPS stimulation showed no difference between WT and *Gnaq*^*−/−*^ Bregs (see Additional file 1: Figure S1E). In order to address whether Gαq has a role in regulating Breg differentiation, splenic B cells were isolated from WT and *Gnaq*^*−/−*^ mice, and then stimulated with lipopolysaccharide (LPS) for 48 h, which was shown to induce IL-10^+^ Breg differentiation [4]. After stimulation, the percentage of CD19^+^IL-10^+^ Bregs was significantly lower in *Gnaq*^*−/−*^ mice (Fig. 1g, h). In addition, the expression of IL-10 in culture supernatant was also lower in *Gnaq*^*−/−*^ mice when compared with that in WT mice (Fig. 1i). The IL-17 expression was slightly decreased in both groups, which might due to Breg differentiation by LPS stimulation (see Additional file 1: Figure S1F). Due to a critical role of IL-35 and TGF-β in Breg suppressive function, we also detected the expression of IL-35 and TGF-β. TGF-β production was lower in *Gnaq*^*−/−*^ mice, while IL-35 expression was comparable with that in WT mice (see Additional file 2: Figure S2). Additionally, the inhibitory ligand PD-L1 on *Gnaq*^*−/−*^ Bregs was also decreased. No different change was observed in FasL expression (Fig. 1j, k).

**Fig. 2:**
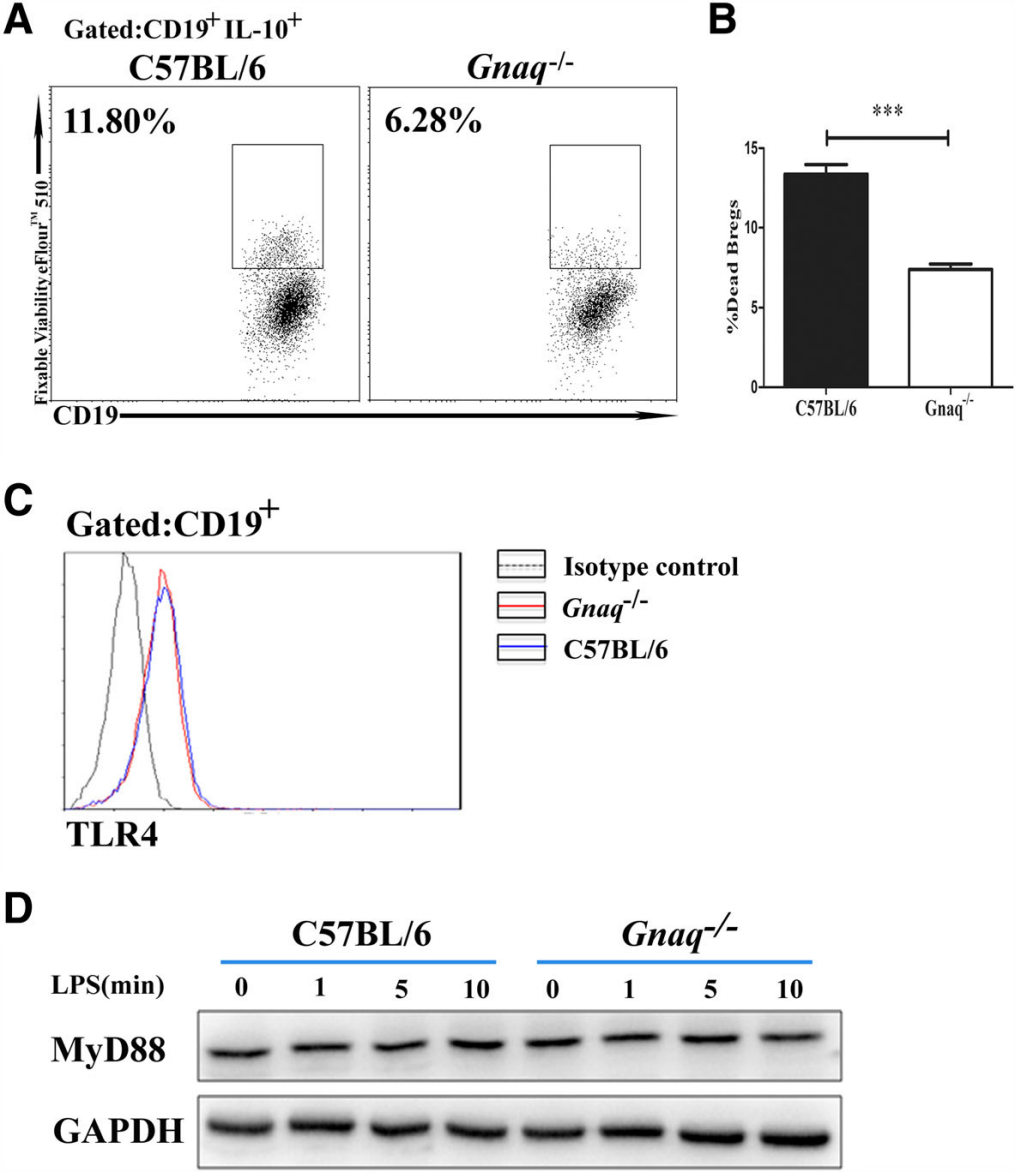
Gαq deficiency did not affect TLR4 signaling in B cells. **a**, **b** B cells purified from WT and *Gnaq*^*−/−*^ mice stimulated with LPS for 48 h, and PMA, ionomycin, and BFA added for last 5 h. Fixable Viability Dye eFlour™ 510 used to analyze B-cell death by flow cytometry. Data presented as mean ± SD of five mice. Results shown are one from three independent experiments. Student’s *t* test analyzed statistical difference. ***p < 0.001. **c** Splenic cells from WT and *Gnaq*^*−/−*^ mice stained with anti-mouse CD19 and anti-mouse TLR4, followed by flow cytometry analysis (*n* = 5). Representative histogram shown. Blue line, WT mice; red line, *Gnaq*^*−/−*^ mice. Data representative of three independent experiments. **d** WT and *Gnaq*^*−/−*^ B cells treated with LPS, then harvested to analyze Myd88 expression by western blot analysis at indicated times. Data representative of three independent experiments. Breg regulatory B cell, IL interleukin, LPS lipopolysaccharide, MyD88 myeloid differentiation primary response 88, TLR Toll-like receptor

To rule out the effect of cell death on the decreased percentages of *Gnaq*^*−/−*^ Bregs after 48 h LPS stimulation, the rates of dead cells were analyzed in the WT and *Gnaq*^*−/−*^ Bregs. We found that Gαq deficiency did not promote cell death, while a decreased rate of cell death in *Gnaq*^*−/−*^ Bregs was observed (Fig. 2a, b), which was in keeping with our previous published data [24]. Previous studies have shown the role of Toll-like receptors (TLRs) in B cell-mediated regulation [28–30]. Since we induced Bregs using LPS, the agonist of TLR4, we next detected TLR4 expression on both WT and *Gnaq*^*−/−*^ B-cell surfaces. As shown in Fig. 2, there was no difference between them. Furthermore, myeloid differentiation primary response 88 (MyD88), a key signaling molecule downstream of TLRs, was also analyzed by immunoblotting. Consistently, no marked differences were observed (Fig. 2c, d). These data indicated that Gαq has no effect on the TLR4/MyD88 signal pathway.

### Gαq is required for Breg immunosuppression

**Fig. 3:**
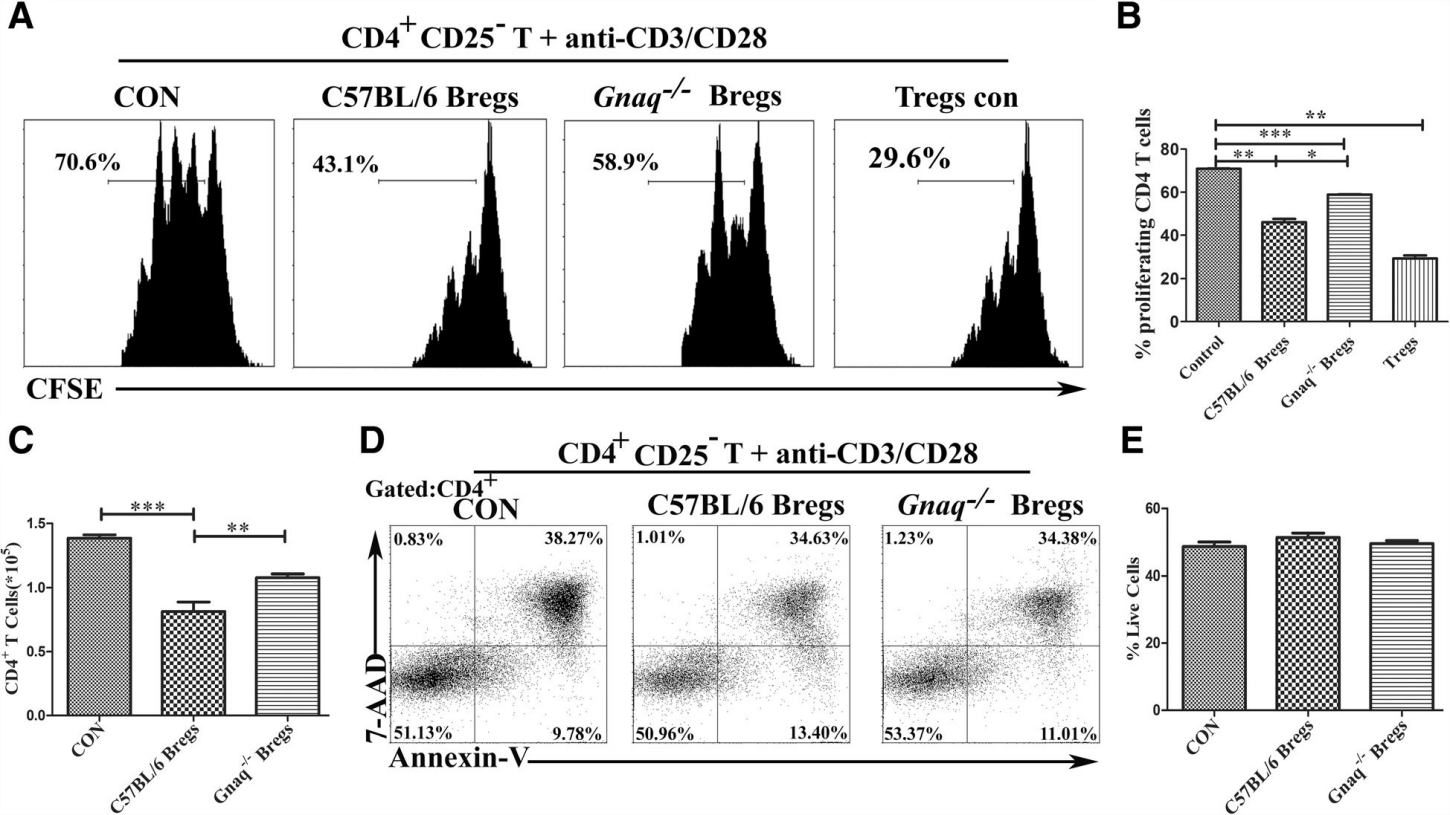
Lack of Gαq impairs ability of Bregs to suppress CD4^+^ T-cell proliferation. CFSE-labeled WT CD4^+^CD25^−^ T cells cocultured (1:1) with sorting-purified WT Bregs, *Gnaq*^*−/−*^ Bregs, or WT Tregs in presence of Mouse T-Activator CD3/CD28 Dynabeads™ for 72 h. Percentage of proliferated CD4^+^ T cells detected by flow cytometry (**a**, **b**), and total number of T cells also investigated (**c**). **d**, **e** Culture cells also harvested to analyze viability of T cells. Cells labeled with anti-mouse CD4, Annexin-V, and 7-AAD, and then analyzed by flow cytometry. Results represent mean ± SD per group (*n* = 5 mice/group). Data representative of three independent experiments. Student’s *t* test analyzed statistical difference. *p < 0.05, **p < 0.01, ***p < 0.001. Breg regulatory B cell, CON control, Treg regulatory T cell

**Fig. 4:**
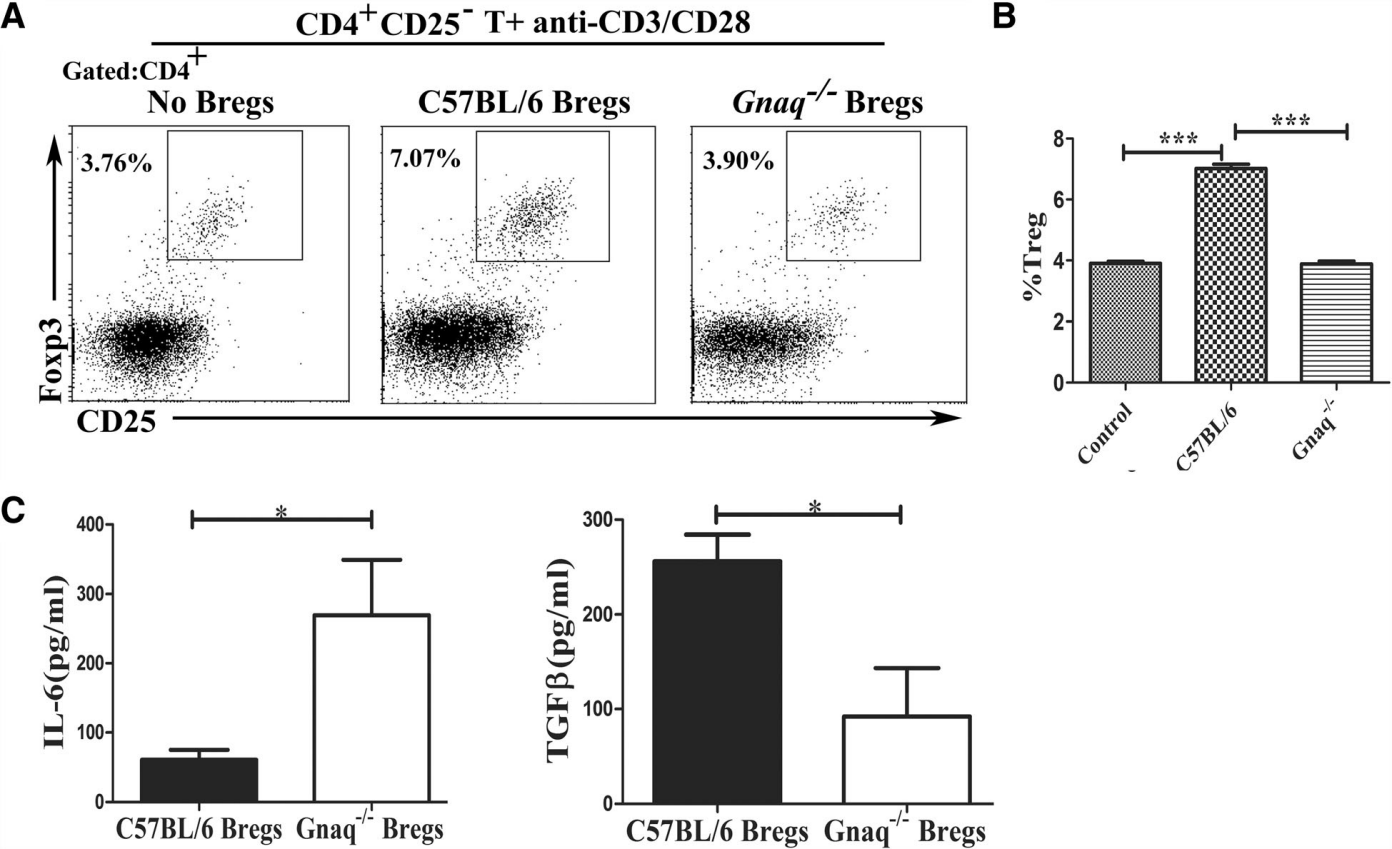
Loss of Gαq in Bregs reduces Tregs in vitro. CD19^+^CD1d^hi^CD5^+^ Bregs isolated from WT or *Gnaq*^*−/−*^ mice and cocultured with purified CD4^+^CD25^−^ T cells from spleen of WT mice for 72 h in presence of Mouse T-Activator CD3/CD28 Dynabeads™. After 72 h, cells and culture supernatants harvested. **a**, **b** Cells stained with anti-CD4, anti-CD25, and anti-Foxp3 antibody to analyze Tregs. **c** Supernatants harvested for ELISA analysis of IL-6 and TGF-β. Results represent mean ± SD per group (*n* = 5 mice/group). Data representative of three independent experiments. Student’s *t* test analyzed statistical difference. *p < 0.05, ***p < 0.001. Breg regulatory B cell, IL interleukin, TGF-β transforming growth factor beta, Treg regulatory T cell

To verify whether *Gnaq*^*−/−*^ Bregs have an inhibitory effect on T-cell proliferation, CD1d^hi^CD5^+^ B cells were sorted from both WT and *Gnaq*^*−/−*^ mice, and then we cocultured with purified CD4^+^CD25^−^ T cells from the WT mice for 72 h under the stimulation of Mouse T-Activator CD3/CD28 Dynabeads™. Although it was weaker than that of Tregs, the inhibitory effect of WT Bregs on T-cell proliferation was significantly strong when compared to that of *Gnaq*^*−/−*^ Bregs (Fig. 3a–c). Furthermore, there was no difference among control Bregs, WT Bregs, and *Gnaq*^*−/−*^ Bregs in the viability of T cells (Fig. 3d, e), which indicated that the different T-cell proliferation was not due to different cell apoptosis. Previous studies have revealed that Bregs can restrain inflammation by promoting differentiation of Tregs [6, 7]. To evaluate the contribution of Gαq in this function of Bregs, we cocultured Bregs purified from WT or *Gnaq*^*−/−*^ mice with WT CD4^+^CD25^−^ T cells. As expected, an increased frequency of CD4^+^CD25^+^Foxp3^+^ Tregs was detected in the WT group after stimulation, whereas there was no significant change of Foxp3 expression in the *Gnaq*^*−/−*^ group when compared with the no Breg experimental group (Fig. 4a, b). Lots of studies have demonstrated that the cytokines IL-6, IL-23, and TGF-β act a crucial role in the regulation of Treg differentiation. Next, we analyzed these cytokines in the supernatants of cocultured WT or *Gnaq*^*−/−*^ Bregs with activated CD4^+^CD25^−^ T cells. As expected, IL-6 was increased in *Gnaq*^*−/−*^ Bregs, whereas TGF-β production was decreased (Fig. 4c). Unfortunately, the IL-23 concentration was lower than the sensitivity of the test kit. These data showed that Gαq modulated Breg immunosuppression by regulating Breg cytokine production, which might affect Treg differentiation.

### Involvement of PI3K, Erk1/2, and p38 MAPK pathways in the regulation of Gαq on Breg differentiation

**Fig. 5:**
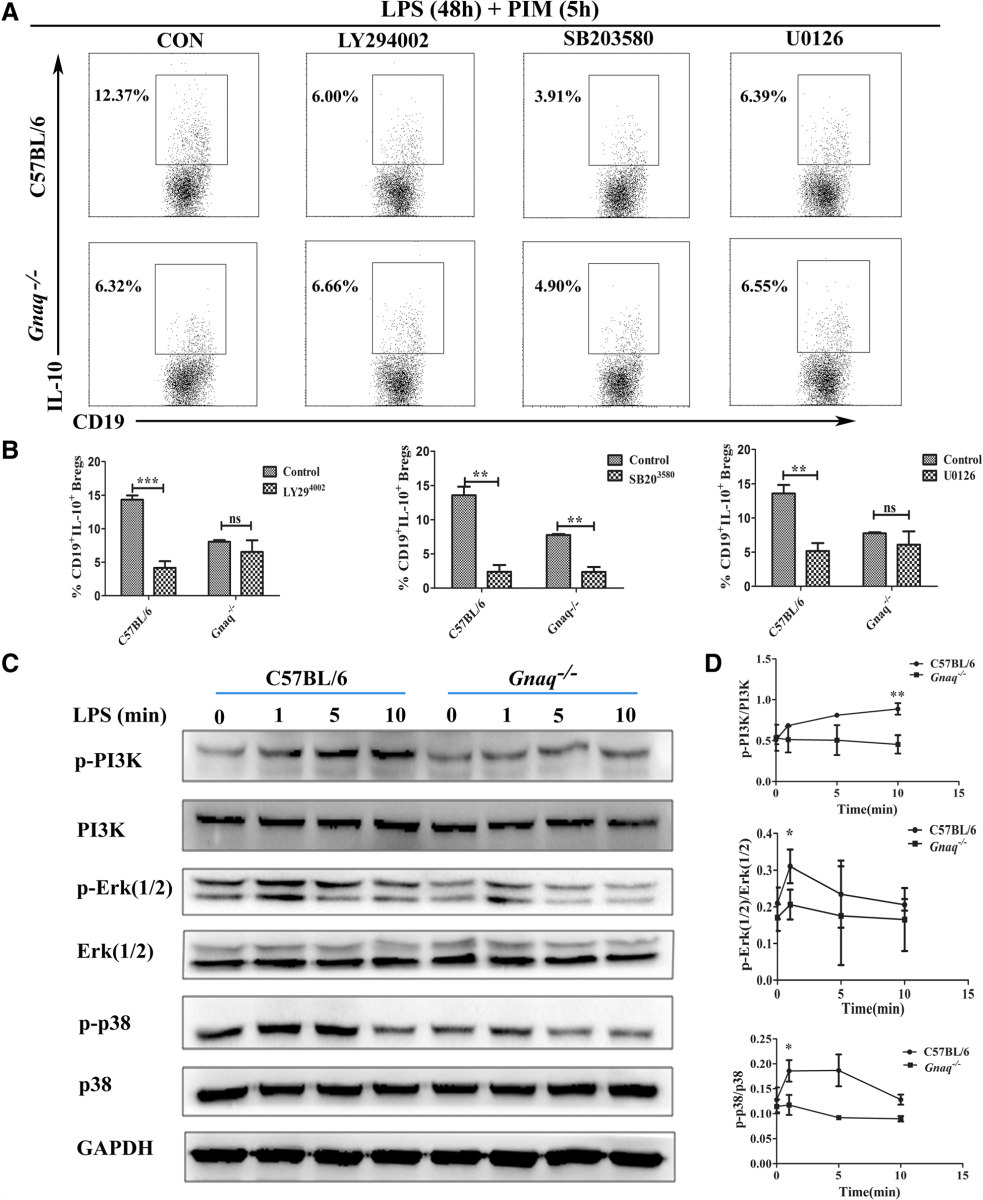
Decreased activation of PI3K, Erk1/2 MAPK, and p38 MAPK signaling pathways in *Gnaq*^*−/−*^ Bregs. Isolated B cells from WT and *Gnaq*^*−/−*^ mice cultured in presence of SB203580 (p38 inhibitor) (2.65 μM), U0126 (Erk1/2 inhibitor) (26 μM), or LY294002 (PI3K inhibitor) (6.4 μM) for 1 h, then cells stimulated by LPS for 48 h, and PMA, ionomycin, and brefeldin A added for last 5 h of culture. After culture, cells stained with anti-mouse CD19 and intracellular staining with IL-10, followed by flow cytometry analysis (**a**, representative images; **b**, statistical analysis). **c** Splenic B cells purified from WT and *Gnaq*^*−/−*^ mice and stimulated with LPS for 0–10 min. Protein from cell lysates exacted for analysis of phospho-PI3K, PI3K, phospho-Erk1/2, Erk1/2, phospho-p38 MAPK, and p38 MAPK by western blot analysis with specific antibodies individually. GAPDH used as loading control. **d** Protein expression levels quantified with Image Lab software. Ratios of phosphor-specific proteins versus total proteins obtained. Results represent mean ± SD per group (*n* = 4–5 mice/group). Data representative of three independent experiments. Student’s *t* test analyzed statistical difference. *p < 0.05, ***p < 0.001, ***p < 0.001. Breg regulatory B cell, CON control, Erk1/2 extracellular regulated protein kinases 1/2, GAPDH glyceraldehyde 3-phosphate dehydrogenase, IL interleukin, LPS lipopolysaccharide, ns not significant, PI3K PI3 kinase, PIM PMA/ionomycin/brefeldin A

Numerous reports indicate that Gαq was implicated in regulating the MAPK pathways, PI3K/Akt pathways, and PLC-β activation [15, 31]. Interestingly, these pathways were also involved in production of IL-10 [32]. Therefore, we supposed that there might be crosstalk between Gαq and IL-10 signaling pathways. To address this hypothesis, we first confirmed these signal pathways in Breg differentiation. The differentiation of WT Bregs was significantly decreased in the presence of LY294002 (PI3K inhibitor), U0126 (Erk1/2 inhibitor), or SB203580 (p38 MAPK inhibitor). However, no significant changes were observed in *Gnaq*^*−/−*^ Bregs (Fig. 5a, b), which may be related to a significantly reduced activation of these signaling pathways in *Gnaq*^*−/−*^ Bregs. Consistently, we found that the levels of phospho-PI3K, phospho-Erk1/2, and phospho-p38 MAPK were lower after LPS stimulation in *Gnaq*^*−/−*^ B cells when compared with those in WT B cells (Fig. 5c, d). In conclusion, the decreased activation of PI3K, Erk1/2, and p38 MAPK contributed to the impaired differentiation of *Gnaq*^*−/−*^ Bregs.

### Decreased frequency of CD19^+^CD24^hi^CD38^hi^ Bregs was correlated with Gαq mRNA expression in RA patients

**Fig. 6:**
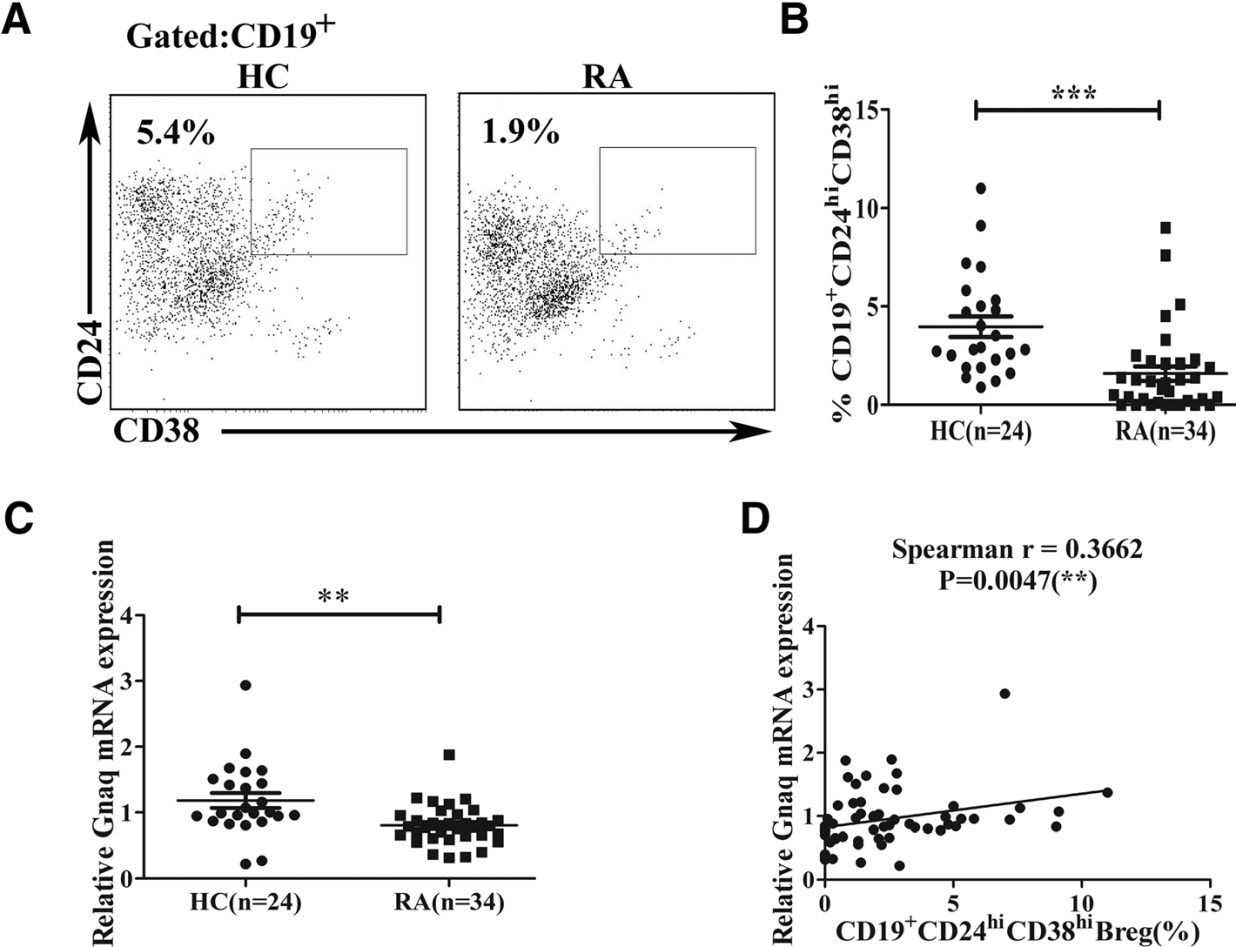
Correlation of frequencies of CD19^+^CD24^hi^CD38^hi^ B cells with Gαq mRNA expression in PBMCs from RA patients. PBMCs isolated from patients with RA and healthy individuals stained with CD19, CD24, and CD38. Representative flow cytometry plots showed CD19^+^CD24^hi^CD38^hi^ B-cell subpopulations in PBMCs from healthy individuals (*n* = 24) and RA patients (*n* = 34) (**a**, representative images; **b**, statistical analysis). **c** Expression level of Gαq assessed by qPCR and normalized to β-actin in PBMCs from HC and RA patients. Mann–Whitney test compared data between two groups. **d** Correlation coefficient between Gαq expression and CD19^+^CD24^hi^CD38^hi^ B-cell frequencies analyzed using the Spearman test (*n* = 58). **p < 0.01, ***p < 0.001. Breg regulatory B cell, HC healthy controls, RA rheumatoid arthritis

Our presented experiments demonstrated that Gαq exerted a role in regulating Bregs in mice. Previous studies have shown that the frequency of CD19^+^CD24^hi^CD38^hi^ Bregs was decreased in RA patients. Human CD19^+^CD24^hi^CD38^hi^ Breg subsets have been shown to maintain tolerance in immune disorders via the release of IL-10 [9]. Here, to further address the regulation of Gαq in Breg function, we analyzed the correlation between Gαq mRNA expression and frequency of regulatory B cells in PBMCs from RA patients. In comparison to HC, a decreased frequency of CD19^+^CD24^hi^CD38^hi^ B cells was observed in RA patients (Fig. 6a, b), and the mRNA expression of Gαq was also significantly lower in the RA group (Fig. 6c). Consistent with the animal results, we observed a significant positive correlation between the frequency of CD19^+^CD24^hi^CD38^hi^ B cells and the expression of Gαq mRNA in PBMCs from patients with RA and HC (Fig. 6d). These data further confirm that Gαq plays a critical role in immune tolerance via regulation of Bregs.

## Discussion

Recent studies have shown that Bregs play a crucial role in autoimmune diseases through suppressing the differentiation of Th1 and Th17 cells, and promoting Treg induction [9]. However, the mechanism of Breg differentiation still remains unknown. Our previous studies demonstrated that Gαq exerts an important role in immune regulation, including Th1 and Th17 function, while the role of Gαq in Breg regulation is still unclear. Here, we found that the differentiation and immunosuppressive effect of Bregs were inhibited in the *Gnaq*^*−/−*^ mice. In addition, our data demonstrated that the PI3K, Erk1/2, and p38 MAPK signaling pathways were involved in the regulation of Breg function by Gαq. Furthermore, we also showed a decreased frequency of CD19^+^CD24^hi^CD38^hi^ Bregs in RA patients, which positively correlated with Gαq mRNA expression. These data suggest that Gαq was involved in the immune tolerance via regulating Breg function.

The existence of B cells with a suppressive capacity was initially reported in the study of guinea pigs in the mid-1970s [33, 34]. In the past 40 years, lots of studies have been focused on regulatory B cells and their mechanisms of action. Mizoguchi et al. [35] defined the B cells that produce IL-10 as regulatory B cells. Through producing IL-10, IL-35, and TGF-β, Bregs suppress immunopathology by prohibiting the expansion of pathogenic T cells and maintaining the pool of Tregs [9]. In our study, we also showed that the impaired immunosuppression of *Gnaq*^*−/−*^ Bregs might be due to the decreased production of IL-10 and TGF-β. Bregs have been considered an important immune regulatory cell in many diseases, such as EAE, type 1 diabetes, collagen-induced arthritis, inflammatory bowel diseases, lupus, and so on [36]. Similarly, CD19^+^CD24^hi^CD38^hi^ B cells, which were considered Bregs in human, can limit the differentiation of naïve CD4^+^ T cells into Th1 and Th17 populations, and maintain Treg function [7]. RA patients with active disease have reduced numbers of CD19^+^CD24^hi^CD38^hi^ B cells in PBMCs compared with healthy individuals [7]. Our data also found a remarkable decrease in the frequency of CD19^+^CD24^hi^CD38^hi^ Bregs in RA patients. Although the number of CD19^+^CD24^hi^CD38^hi^ B cells was increased in SLE patients, they lacked the suppressive capacity due to their failure to produce IL-10 [12]. Previous studies showed that IL-10 production of human B cells was associated with the activation of STAT3 and ERK [37]. Our current findings showed that IL-10 production was also impaired in Bregs in the absence of Gαq.

Activation of the ERK pathway is a common requirement for IL-10 expression by T cells, macrophages, and myeloid dendritic cells [32]. Abrogation of either ERK or p38 activation after TLR stimulation leads to a reduced IL-10 expression, which suggests that these two pathways might cooperate in TLR-induced IL-10 production [32]. Consistently, inhibition of PI3K, Erk1/2, or p38 MAPK significantly ablates the Breg differentiation in our study here. As expected, we found that the basal levels of phospho-PI3K, phospho-Erk1/2, and phospho-p38 MAPK in response to LPS were lower in *Gnaq*^*−/−*^ B cells than in WT B cells. These data suggest that Gαq was involved in the differentiation of Bregs partly through regulation of PI3K, Erk1/2, or p38 MAPK signaling. That IL-10 can be induced by LPS in many cells has been demonstrated. However, we here observed no marked differences of TLR4 and MyD88 expression between B cells from WT and *Gnaq*^*−/−*^ mice, which further confirms the regulation of Gαq in the PI3K, Erk1/2, and p38 MAPK signaling pathways of Breg function.

In a previous study we demonstrated that *Gnaq*^*−/−*^ chimeras could spontaneously develop manifestations of systemic autoimmune disease with high titer antinuclear antibody and inflammatory arthritis, and B cells from *Gnaq*^*−/−*^ mice have an intrinsic survival advantage over normal B cells, suggesting that Gαq is critically important for maintaining control of peripheral B-cell tolerance induction and repressing autoimmunity [24]. However, the role of Gαq in Breg regulation remains unknown. Actually, the percentage of Bregs was significantly lower in the spleen of *Gnaq*^*−/−*^ mice. Consistent with the animal experiments, our data here showed a significant positive correlation between the frequency of CD19^+^CD24^hi^CD38^hi^ Bregs and the expression of Gαq mRNA in PBMCs from patients with RA and HC. Our current findings showed that Gαq deficiency limited the differentiation of Bregs. Several studies demonstrated that Bregs were important for the generation and maintenance of Tregs [4]. Bregs could induce the differentiation of type 1 regulatory T (Tr1) cells [38, 39]. Moreover, Bregs might promote the differentiation of other type of regulatory T-cell subsets [40]. Consistent with prior studies, purified WT Bregs could convert CD4^+^CD25^−^ T cells into Tregs, but this function of *Gnaq*^*−/−*^ Bregs was impaired. Indeed, we also observed an impaired inhibition of T-cell expansion in *Gnaq*^*−/−*^ Bregs. This might be the reason for impaired suppressive function of *Gnaq*^*−/−*^ Bregs on T-cell proliferation. Some studies suggested that CD40 mAb-stimulated CD1d^hi^CD5^+^ B cells could not regulate T-cell proliferation in vitro [41]. TLRs and CD40 activation are well-characterized signals in Breg differentiation [4, 9]. However, LPS but not CD40 activator can induce IL-10 secretion [36], which might be the reason for no effect on T-cell proliferation inhibition being observed.

## Conclusions

Although we do not yet know whether Gαq deficiency in Bregs alone is sufficient to induce autoimmune disease, our work showed a critical intrinsic role for Gαq in the maintenance of Breg differentiation and function. Furthermore, our data suggested that the regulation of Gαq on Breg differentiation might occur partly via the PI3K, Erk1/2, and p38 MAPK signaling pathways. Our study here provides new insights into the mechanisms of Breg immune tolerance.

## Additional files

Additional file 1: **Figure S1.** Gαq deficiency promoted B cells to secrete inflammatory cytokines. (**A–D**) Splenic cells isolated from *Gnaq*^*−/−*^ mice and WT littermates and subjected to flow cytometry analysis after PMA, ionomycin, and BFA stimulation. Splenic cells stained with anti-mouse CD19, followed by intracellular staining with IFN-γ and IL-17a. Representative images and statistical analysis of CD19^+^IFN-γ^+^ and CD19^+^IL-17a shown in (A, C) and (B, D) respectively. (**E**) Splenic B cells purified from WT and *Gnaq*^*−/−*^ mice and stimulated with LPS for 0–10 min. Protein from cell lysates exacted and analyzed using western blot analysis. Phospho-STAT1 and STAT1 probed using specific antibodies individually. β-Tubulin used as control protein. (**F**) B cells isolated from the spleens of WT and *Gnaq*^*−/−*^ mice and stimulated with LPS for 48 h, and then PMA, ionomycin, and BFA added for last 5 h. After culture, cells stained with anti-mouse CD19, followed by intracellular staining with IL-17a. Results represent mean ± SD per group (*n* = 6–8 mice/group). Student’s *t* test analyzed statistical difference. Data representative of three independent experiments. *p < 0.05, **p < 0.01 (DOCX 1303 kb)

Additional file 2: **Figure S2.** Gαq deficiency impaired anti-inflammatory cytokines in Bregs. B cells isolated from spleen of WT and *Gnaq*^*−/−*^ mice and stimulated with LPS for 48 h, and then culture supernatant was harvested and subjected to analyze levels of IL-35 (**A**) and TGF-β (**B**) by ELISA. Results represent mean ± SD per group (*n* = 6–8 mice/group). Student’s *t* test analyzed statistical difference. Data representative of three independent experiments. **p < 0.01 (DOCX 1000 kb)

## Abbreviations

Breg: Regulatory B cell
EAE: Experimental autoimmune encephalitis
Erk1/2: Extracellular regulated protein kinases 1/2
G protein: Guanine nucleotide-binding protein
GPCR: G protein-coupled receptor
HC: Healthy controls
IL: Interleukin
LPS: Lipopolysaccharide
MyD88: Myeloid differentiation primary response 88
p38 MAPK: p38 mitogen-activated protein kinase
PBMC: Peripheral blood mononuclear cell
PI3K: PI3 kinase
qPCR: Quantitative real-time PCR
RA: Rheumatoid arthritis
SD: Standard deviation
SLE: Systemic lupus erythematosus
TGF-β: Transforming growth factor beta
Th1: T helper 1
Th17: T helper 17
TLR: Toll-like receptor
Treg: Regulatory T cell
WT: Wild type
