# Supplementary figures and images for: Loss of Gαq impairs regulatory B-cell function
Source: Arthritis Res Ther. 2018 Aug 24;20:186. doi: 10.1186/s13075-018-1682-0 (PMC6109260; doi:10.1186/s13075-018-1682-0)

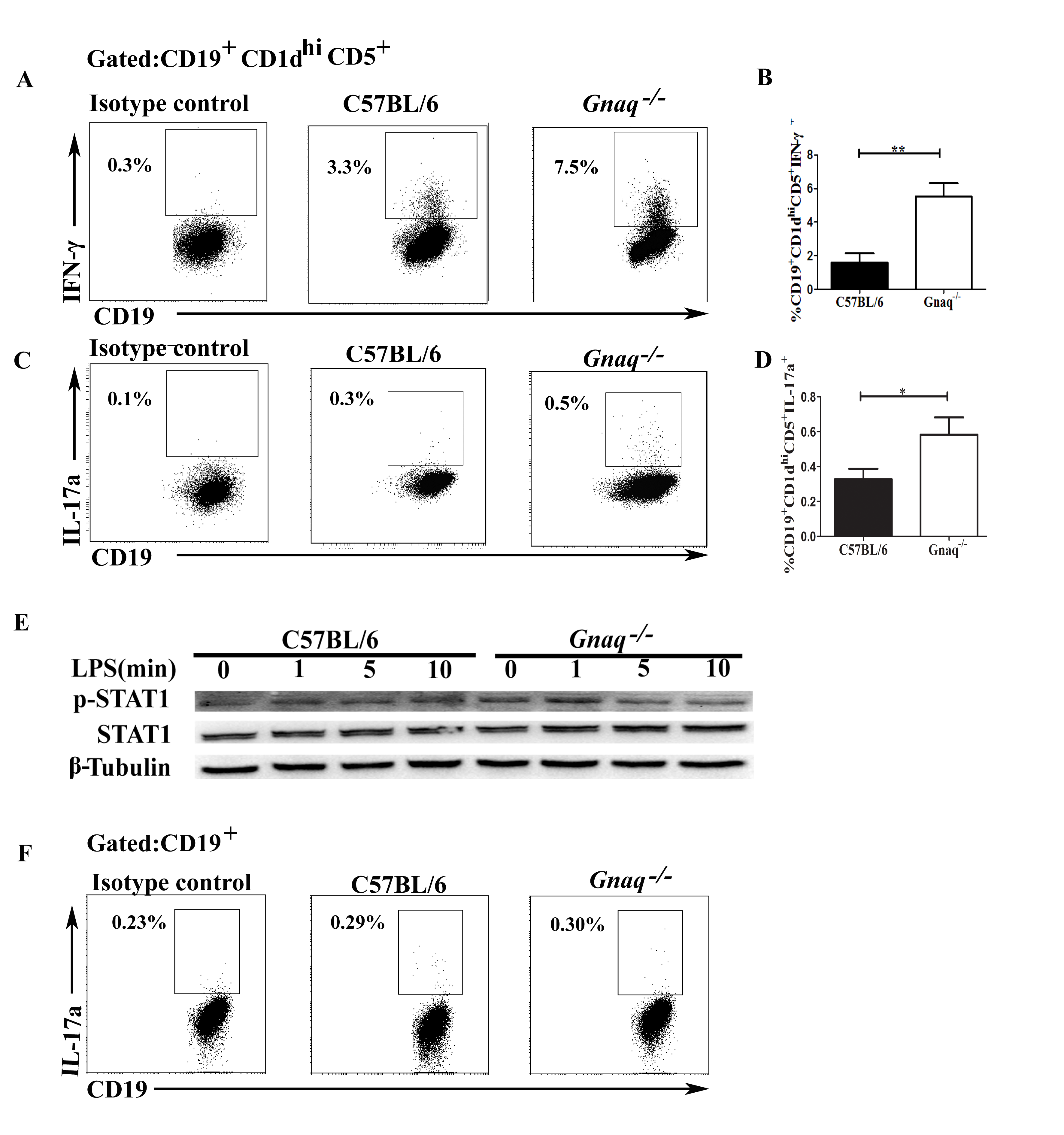

Supplement: Supplementary file 1 — Figure S1. Gαq deficiency promoted B cells to secrete inflammatory cytokines. (A–D) Splenic cells isolated from Gnaq−/− mice and WT littermates and subjected to flow cytometry analysis after PMA, ionomycin, and BFA stimulation. Splenic cells stained with anti-mouse CD19, followed by intracellular staining with IFN-γ and IL-17a. Representative images and statistical analysis of CD19+IFN-γ+ and CD19+IL-17a shown in (A, C) and (B, D) respectively. (E) Splenic B cells purified from WT and Gnaq−/− mice and stimulated with LPS for 0–10 min. Protein from cell lysates exacted and analyzed using western blot analysis. Phospho-STAT1 and STAT1 probed using specific antibodies individually. β-Tubulin used as control protein. (F) B cells isolated from the spleens of WT and Gnaq−/− mice and stimulated with LPS for 48 h, and then PMA, ionomycin, and BFA added for last 5 h. After culture, cells stained with anti-mouse CD19, followed by intracellular staining with IL-17a. Results represent mean ± SD per group (n = 6–8 mice/group). Student’s t test analyzed statistical difference. Data representative of three independent experiments. *p < 0.05, **p < 0.01 (DOCX 1303 kb) [file 13075_2018_1682_MOESM1_ESM.docx]

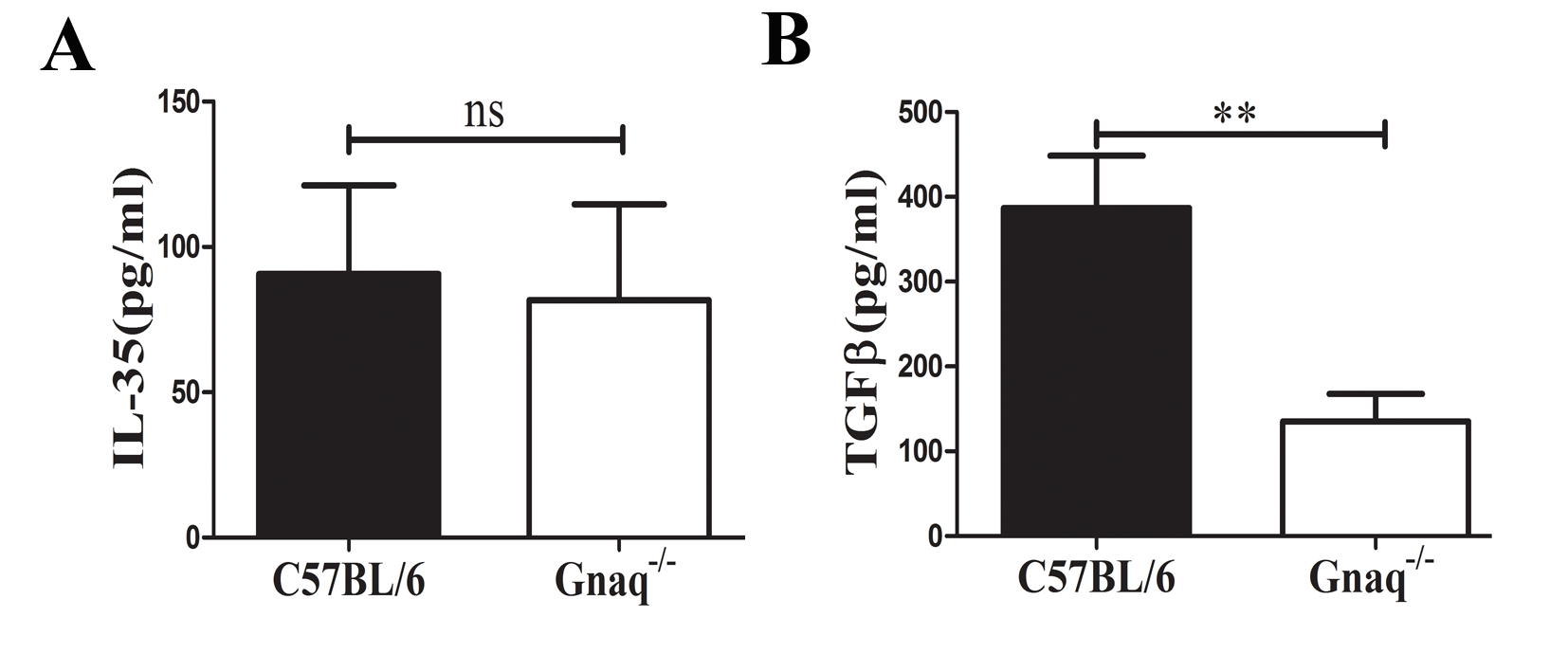

Supplement: Supplementary file 2 — Figure S2. Gαq deficiency impaired anti-inflammatory cytokines in Bregs. B cells isolated from spleen of WT and Gnaq−/− mice and stimulated with LPS for 48 h, and then culture supernatant was harvested and subjected to analyze levels of IL-35 (A) and TGF-β (B) by ELISA. Results represent mean ± SD per group (n = 6–8 mice/group). Student’s t test analyzed statistical difference. Data representative of three independent experiments. **p < 0.01 (DOCX 1000 kb) [file 13075_2018_1682_MOESM2_ESM.docx]
